# PhytoSynthesized Nano-Zinc Formulation for Sustainable Growth and Yield Enhancement in Tomatoes Under Protected Cultivation

**DOI:** 10.3390/plants15182862

**Published:** 2026-09-18

**Authors:** Lekkala Venkata Ravishankar, VijayaDurga V. V. Lekkala, Dakshayani Lomada, Madhava C. Reddy, Nidhi Puranik, Shraddha Tiwari

**Affiliations:** 1 Production and Plantations, Red Otter Farms Pvt Ltd., Uttarakhand 263159, India; lvrsrreddy@gmail.com; 2 Department of Genetics and Genomics, Yogi Vemana University, Kadapa 516005, India; lvijayadurga123@gmail.com (V.V.V.L.); dlomadayvu@gmail.com (D.L.); 3 Department of Biotechnology and Bioinformatics, Yogi Vemana University, Kadapa 516005, India; 4 Department of Life Sciences, Yeungnam University, Gyeongsan 38541, Republic of Korea; nidhipuranik30@gmail.com

**Keywords:** *Moringa oleifera*, antioxidant, tomato, micronutrient, foliar application, zinc nanoparticles

## Abstract

Zinc (Zn), an important micronutrient, plays an essential role in plant growth and development. Being the most crucial divalent metallic enzyme, Zn acts as a catalyst in many biochemical processes that regulate growth hormones in plants. Nanoseed priming is an effective agrotechnology that enhances the quality parameters of young seedlings for establishing a good crop stand. In the present study, zinc nanoparticles synthesized from *M. oleifera* leaf extracts were characterized by UV spectroscopy, XRD, FTIR, FE-SEM, TEM, TGA, DLS, and zeta analysis. The synthesized nanoparticles showed moderate antioxidant, anti-inflammatory, and antimicrobial activities. Phytosynthesized nanoparticles were tested for their efficiency in modulating plant growth and development, where modern nanoscience technology is at the seedling stage. In the present research, tomato seeds were hydropriming carried out with sample solution of 100 mL from a 1 L stock solution for each treatment with ZnONPs at 50 ppm, 100 ppm, 150 ppm, 200 ppm, and 250 ppm, as well as ZnSO_4_ used as an ionic control at 1.5 g/L. 150 and 200 ppm increased germination percentage. Plant height at seedling stage was enhanced to 22.33 and 19.67 cm at 150 and 250 ppm, respectively. Foliar applications were initiated at 35 DAT, when the plants were at 10–12 true leaves and in the transition phase of tomato plants from the vegetative stage to the flowering stage; 150 ppm induced early flowering, as well as the highest leaf area, TSS content, chlorophyll levels, the number of fruits per cluster, and the number of fruits per plant. Foliar application at 150 ppm enhanced the total and marketable yields to a maximum of 4102.63 and 3984.30 g, respectively. Foliar application at 150 ppm induced the lowest disease severity, at 20.02%, in vivo and in vitro, with the maximum mycelial inhibition of 19.50 mm at 0.15 mg/mL.

## 1. Introduction

Zinc (Zn), or Zn^+2^ as a divalent cation, is the 23rd most abundant element available in the Earth’s crust, primarily within a blending ore, sphalerite [1]. It is available in all living cells; being a micronutrient in plants, it is widely distributed across tissues and has multifunctional roles at very low concentrations [2,3]. At the molecular level, zinc acts as an integral component of several biomolecules like lipids and proteins, and also as a cofactor for auxins [4]. Zinc plays a critical role in physiological processes such as uptake and metabolism of nitrogen for protein synthesis, chlorophyll biosynthesis, carbon anhydrase activity, resistance to biotic and abiotic stress, and oxidative cell damage [5,6,7]. Zn is the only metal necessary for all six enzyme classes: oxidoreductases, transferases, hydrolases, lyases, isomerases, and ligases [8]. Zinc absorption in plants is an extremely complex phenomenon, mainly governed by transporters and metal chelators. Subcellular studies document that Zn hyperaccumulators participate via specific Zn transporters, vacuolar sequestration, and detoxification mechanisms for maintaining Zn homeostasis [9]. Zinc plays a dual role in crop growth and productivity and in protection by regulating resistance to different biotic stressors like fungi, bacteria, and oomycetes [10]. Foliar application of zinc is important, particularly in solanaceous vegetables, for increasing yield and nutritional parameters [11,12]. Reducing the particle size of any element helps in achieving application on the target site rather than causing the buildup of spray droplets; the particle size must be between 1 and 100 nm [13].

*Moringa oleifera* (Moringaceae) is broadly distributed over Southeast Asia and Africa. The *Moringa* genus consists of 13 species [14,15]. Some countries consume edible parts due to their nutritional value [16]. *Moringa oleifera* is a storehouse for nutrients and minerals like potassium, zinc, iron, magnesium, and copper, as reported by Kasolo et al. The phytoconstituents comprise enzymes and vitamins [17,18], and various secondary metabolites, including steroids, tannins, saponins, terpenoids, and alkaloids [19]. Leaf extracts contain several nutrients, including vitamins, carotene, amino acids, and minerals [17]. Numerous parts of plants—flowers, leaves, seeds, and fruits—are rich sources of protein, phenolics, minerals, and amino acids. Due to its potential therapeutic benefits, it is considered an essential herb in Ayurvedic medicine to cure heart issues. It is also used to treat fungal infections as it possesses antifungal properties. *M. oleifera* possesses several biological and pharmacological properties, such as anticancer, antioxidant, anti-inflammatory, antipyretic, antitumor, antidiuretic, antihypersensitivity, hypolipidemic, and hypoglycemic effects; it lowers cholesterol levels and has a hepatoprotective influence [20,21]. Additionally, it is used against filariasis, malaria, schistosomiasis, and trypanosomiasis, suggesting its antiparasitic activities [22].

Nanotechnology has started garnering more attention regarding health and medical care in the 21st century [23]. Nanomedicine has a high impact on health care by providing breakthroughs in many scientific bottlenecks [24,25,26]. Nanoparticles have unique and remarkable characteristics due to their small size, shape, and high particle distribution, which have numerous biomedical applications. The design and development of nanoparticles has led to their use in countless fields, including sensing, diagnostic imaging, and cancer therapies [26,27]. Diverse nanoparticle production methods are available, including biological, chemical, and physical methods. Chemical synthesis is costly and involves complex processes such as the absorption of harsh molecules onto the surface, causing hazards in medical applications [28,29,30]. Conversely, the green synthesis approach is cost-effective and less toxic and is achieved using plants, bacteria, algae, and fungi [31,32,33,34] as natural-derived substitutes. In green synthesis, extracts containing various 2° metabolites—flavonoids, alkaloids, ascorbic acid, phenolic compounds, and glycosides—act as reducing agents for producing biogenic nanoparticles [35].

Numerous NPs produced by green approaches, such as silver, gold, zinc oxide, and copper oxide, have been used for several biological studies. Green synthesis holds promise in the manufacture of anticancer and antimicrobial agents that are more specific, economical, and safe for drug delivery [36,37,38,39,40]. Zinc is an essential element and the most crucial cofactor in the body’s metabolism of several proteins, nucleic acids, and hematopoiesis. At the nanoscale, zinc oxide nanoparticles (ZnONPs) exhibit unique properties, such as small shape and size, and can be employed as additives in the food industry. Recommended as harmless and approved by the Food and Drug Administration, these are safe for animals and humans [41,42]. ZnONPs are highly significant metal oxides, and their applications are increasing due to their unique physical and chemical properties [43]. Because of their high UV-adsorption properties, they are used in sunscreen and cosmetic products [44]. Moreover, the nanoscale provides properties such as a wide gap, enhanced transparency, and high electron mobility, making them a good semiconductor material [45,46]. ZnONPs have been utilized in dietary products to enhance growth performance, immune responses, and antioxidant properties [47].

The present research reveals the importance of *M. oleifera*-derived micronutrients under nanoformulations for greater efficacy with target-oriented precise applications for promoting growth and yield-attributing characteristics in tomatoes. It also revealed that the antimicrobial and antifungal properties of zinc nanoparticles obtained through phytomediated synthesis decrease the need for bulk application of synthetic inorganic fertilizers in crop production and protection.

## 2. Materials and Methods

### 2.1. Collection and Preparation of Moringa Oleifera Leaf Extract

*M. oleifera* leaves were collected from Rayachoty, Annamayya district, Andhra Pradesh, India. They were thoroughly washed with tap water followed by distilled water to remove impurities, dried at room temperature, and ground into a smooth powder. Then, 10 g of the powder was added to 100 mL of distilled water and boiled for 20 min in a water bath at 50 °C. The dark greenish-colored solution formed was cooled to room temperature, stored, and used for experiments.

### 2.2. Synthesis of Zn Nanoparticles

Zinc acetate dihydrate was dissolved in 49 mL of distilled water to obtain a 0.02 M solution; 1 mL of aqueous leaf extract was added, and the pH was adjusted to ±10. This mixture was boiled at 70–90 °C while continuously stirring at 300 rpm for 1 h. Finally, the formation of a yellowish-white precipitate indicated the formation of Zinc nanoparticles. Later, the solution was incubated overnight and washed with ethanol twice to remove impurities; a yellowish-white slurry was obtained, which was dried in a hot air oven at 60 °C.

### 2.3. Characterization Techniques

The visible formation of Zn nanoparticles was confirmed by a change in the solution color. Nanoparticles were characterized by UV–Visible (ShimaduUV-1800, Kyoto, Japan) spectral analysis from 250 to 1000 nm. To identify the functional groups involved in ZnONPs synthesis, FTIR spectroscopy (Perkin Elmer, Spectrum 2, Buckinghamshire, UK) was analyzed at wavelengths of 500–4000 cm^−1^ [48]. The surface morphology of the biosynthesized nanoparticles was determined with a Field Emission Scanning Electron Microscope (FE-SEM) (Hitachi High Technologies America Inc., Schaumburg, IL, USA). Samples were dried and coated on the carbon tape surface with gold utilizing a coater. The elemental composition of the NPs was determined using Energy-dispersive X-ray (EDAX, Pleasanton, CA, USA) Spectroscopy. The shape and size distribution of powdered ZnONPs were studied with a transmission electron microscope (TEM) (JEOL, Tokyo, Japan), accelerated at 200 kV. The TEM micrographs were analyzed for size distribution using ImageJ software (Version 1.54p), and a histogram was constructed. The crystalline structure was analyzed using an X-ray diffractometer (Bruker, Billerica, MA, USA). Size and surface charge distribution were determined by Dynamic Light Scattering (DLS) and zeta potential (ζ) analysis.

### 2.4. Evaluation of Antioxidant Activities

Nanoparticles synthesized from *Moringa* leaf extracts were tested for in vitro antioxidant activities using ABTS and DPPH assays. Solutions with different concentrations of standard or nanoparticles were prepared to assay the activity in a concentration-dependent manner. The absorbance was measured spectrophotometrically against blank solutions [42]. The % inhibition was calculated using the formula (Equation (1)).

(1) $$\mathrm{Radical}\;\mathrm{scavenging}\;\mathrm{activity}=\;{OD}_{Control}\;-\;{OD}_{Sample}/{OD}_{Control}\times 100$$

#### 2.4.1. DPPH Radical Scavenging Activity

DPPH radical scavenging activity was determined using a modified Brand-Williams method [49]. Quercetin, a natural flavonoid, was used as a standard to compare the activity of the synthesized nanoparticles [50] in a 96-well microtiter plate. To 100 mL of methanol, 2 mg of DPPH was added and dissolved to obtain a 0.1 mM solution. Different concentrations of ZnONPs were mixed with DPPH solution, incubated in the dark for 30 min, and absorbances were measured at 520 nm [51].

#### 2.4.2. ABTS Radical Scavenging Activity

ABTS scavenging activity was assayed per a protocol adapted from an earlier publication [52]. ABTS^+^ radical solution was prepared by mixing 7 mM ABTS and 2.4 mM ammonium persulfate in 10 mL. The reaction mixture was stored in the dark for 12 h; formation of a dark blue solution indicated the presence of ABTS^+^ radicals. For each experiment, the solution was diluted with methanol to an OD of 0.7 at 734 nm. To determine the ABTS activity of Zinc nanoparticles, different concentrations of ABTS solution—200 μL—were mixed in a 96-well microtiter plate and incubated for 30 min in the dark. The absorbance was taken at 734 nm. Vitamin C served as a standard [53].

### 2.5. Evaluation of Anti-Inflammatory Activities

#### 2.5.1. Protein Denaturation Activity

Protein denaturation activity was ascertained according to a published procedure [54] with minor modifications. Briefly, 200 μL of fresh egg albumin was mixed with 2 mL of PBS containing nanoparticles at concentrations of 25, 50, 75, and 100 μg/mL. The control contained 1 mL of distilled water, PBS and egg albumin. The reaction mixture was incubated at 37 °C for 15 min, and later for 5 min in a water bath at 70 °C. The mixture was allowed to cool, and absorbance was measured at 660 nm. Acetylsalicylic acid served as a standard control [55]. % protein denaturation was calculated by

% Protein Denaturation = (OD_control_ − OD_sample)_ × 100/OD_control_

#### 2.5.2. Membrane Stabilization Method

Preparation of RBC suspension: Blood from a healthy volunteer who had not taken any non-steroidal anti-inflammatory drugs (NSAIDS) for 2 weeks before the experiment was collected in tubes for centrifugation at 3000 rpm for 5 min and then washed three times with saline. Blood volume was measured, and a 10% (*v*/*v*) HRBC suspension was prepared with normal saline [56,57,58].

Hypotonicity-induced hemolysis assay: The control contained 1 mL of PBS, 2 mL of distilled water, and 0.5 mL of HRBC suspension. The test sample contained 2 mL of hyposaline, 0.5 mL of HRBC suspension, and 1 mL of PBS with NPs at concentrations: 25, 50, 75, and 100 μg/mL. Diclofenac was used as a standard drug as a control [59]. The reaction mix was incubated at 37 °C for 30 min and centrifuged at 3000 rpm for 10 min. The absorbance of the supernatant was measured at 560 nm with a spectrophotometer [60]. The % hemolysis was calculated as follows.

% of Haemolysis = (OD_control_ − OD_sample_) × 100/OD_control_

### 2.6. Evaluation of Antibacterial Activities

#### 2.6.1. Antibiofilm Activity

To determine the antibiofilm activities of ZnONPs, 96-well microtiter plates (polystyrene) were used as described earlier [61]. Each well was filled with 100 μL of fresh culture medium and inoculated with the bacteria: *S. aureus*, *E. coli*, *P. aeruginosa*, and *K. pneumoniae*. Subsequently, 10–50 μg/mL of nanoparticles were added; ampicillin served as the standard control. The plate was incubated at 37 °C for 24 h. Later, the contents were discarded, each well was washed once with 1 mL of PBS–saline to remove any adherent bacterial cells, and the plate was air-dried for 30 min. Then, 200 μL of crystal violet was added, re-incubated for 30 min, and washed carefully with water to remove any excess stain. The plate was shade-dried for 30 min, 200 μL of ethanol was added, and finally the absorbance was taken at 590 nm. % antibiofilm activity was measured using the following formula.

% of Biofilm inhibition = (OD_control_ − OD_sample_) × 100/OD_control_

#### 2.6.2. Disk Diffusion Method

Antibacterial activity of ZnONPs was analyzed using the Kirby–Bauer disk diffusion method [62] against MDR strains of *S. aureus*, *E. coli*, *P. aeruginosa*, and *K. pneumoniae* isolated from human wounds [63]. For this experiment, fresh overnight-grown bacterial cultures were spread uniformly on Petri dishes containing Luria–Bertani agar with an L-rod. Sterile paper disks immersed in ZnO-NPs were placed, and diverse concentrations of test samples were added. The plates were incubated at 37 °C for 24 h and observed for zones of inhibition. Ciprofloxacin served as a positive control.

### 2.7. Evaluation of Tomato Plant Growth Parameters

#### 2.7.1. Experimental Site

The study was conducted in a naturally ventilated greenhouse with a closed hydroponic circulation system. The test site, Kotabhag (29.3992° N, 79.3001° E), is situated 1200 m above sea level in Nainital district, Uttarakhand, India.

#### 2.7.2. Experimental Design

The experiment was conducted in a randomized complete block design with nutrient solutions containing different concentrations of ZnONPs, with a constant flow of fertigation through drip irrigation. The treatments are summarized in Table 1; the nutrient mixture was the same for all treatments. The greenhouse was 6 m in height at the center, 20 m in length, and 10 m in width, with 6 separate nutrient tanks, each of 50 L capacity. Each tank was connected to 9 Dutch Bucket systems, with a 60 cm gap between 3 rows for every tank. In total, five pH levels with five ZnONP levels were replicated three times. This study was conducted during the rabi season of 2025–26 under soilless conditions with the recommended fertigation dosages. RO water was used for fertigation under greenhouse conditions. Nurseries were prepared, and seedlings were raised using cocopeat and vermiculite in the ratio of 3:1.

#### 2.7.3. Seed Priming

Due to limited quantity, 10 seeds were used for seed priming under different NP concentrations of 50, 100, 150, 200, and 250 ppm; 0.15 grams/litre ZnSO_4_ solution was used for hydropriming of seeds in 90 mm Petri dishes with 20 mL of experimental solution for each treatment. The seeds were sterilized with 0.1% sodium hypochlorite for 5 min and then submerged under corresponding treatments of 50, 100, 150, 200, and 250 ppm, followed by the germination procedure as described [64].

#### 2.7.4. Seedling Growth Parameters

Nursery growth parameters, such as germination percentage, seedling growth, plant height, root length, internodal length, and plant weight, were calculated 25 days after sowing. These were analyzed before transplanting to cocopeat buckets and upon successful transplanting from the nursery to know the effects of ZnONPs on improving such parameters.

#### 2.7.5. Plant Growth Parameters

Foliar application of different treatments was applied to plants from 35 DAS before the initiation at an interval of 10 days from spraying to another spraying, with a spray volume of 250 mL/plant, for 3 times in each treatment, and the main reason for choosing this period was the positive effects of Zn^+2^ element on flowering; it also elevates the hormonal levels. Before flowering, it was applied to assess how it impacts flowering initiation in these treatments. Days to flowering were calculated to determine the efficacy of ZnONPs under different treatments per an earlier study [65]. Plant height, number of primary branches per plant, and number of fruits per plant were determined. A leaf area meter (LI-3000, Li-COR, Lincoln, NE, USA) was used to calculate the leaf area, represented as cm^2^. Until the last commercial harvest, total fruit weight per plant (kg) was recorded according to a previously described method [14]. The fruit weight in kg per plant was converted into t/ha. Leaf chlorophyll content of each plant of each treatment at the vegetative, flowering, and mature stages was measured using a soil–plant analysis development (SPAD) chlorophyll meter (Konica Minolta, Kowloon, Hong Kong). The SPAD readings were collected from the midribs of fully expanded leaves in each plant. Tomato fruits were harvested from the second week of 15 September 2025 to the end of 31 April 2026. Five fruits from each plant were collected to record fruit length, fruit diameter, and individual fruit weight.

#### 2.7.6. Yield Estimation

Fruits were collected at each harvest from randomly arranged treatments and weighed. The total yield at each harvest was recorded after deducting the harvest wastage [66].

#### 2.7.7. Determination of Quality Parameters

Fresh and mature uniform tomatoes were collected from each treated plant, brought to the laboratory, and preserved by freezing at −30 °C for quality analysis. Total soluble solids (TSS) were measured in fresh fruit juice dropped on the glass lens of a digital pocket refractometer (model PAL1, ATAGOTM Tokyo Tech, Minato City, Japan), and the results were expressed in Brix per an accepted method. Shelf life was calculated by harvesting 80 tomatoes at different ripening stages, and they were stored at room temperature until 50 of the fruit were damaged [67].

#### 2.7.8. Efficacy of ZnONPs on Mitigating Late Blight Infection

Anti-late blight infection efficacy and disease severity (DS) scale were assessed [10]. The DS was calculated under greenhouse conditions that replicated the natural cooler climates where the disease is more prevalent. The efficacy of ZnONPs was evaluated in vitro as per a previous procedure [68].

### 2.8. Statistical Analyses

All experiments were performed at least thrice. Data were statistically analyzed using OriginLab 2018 and Prism 8.4.1 software. Statistical tests included one -way analysis of variance (ANOVA, Kowloon, Hong Kong). The results were expressed as the mean ± SEM, and a *p*-value < 0.05 was considered statistically significant.

## 3. Results

### 3.1. U. V. Visible Spectroscopy Analysis

**Figure 1:**
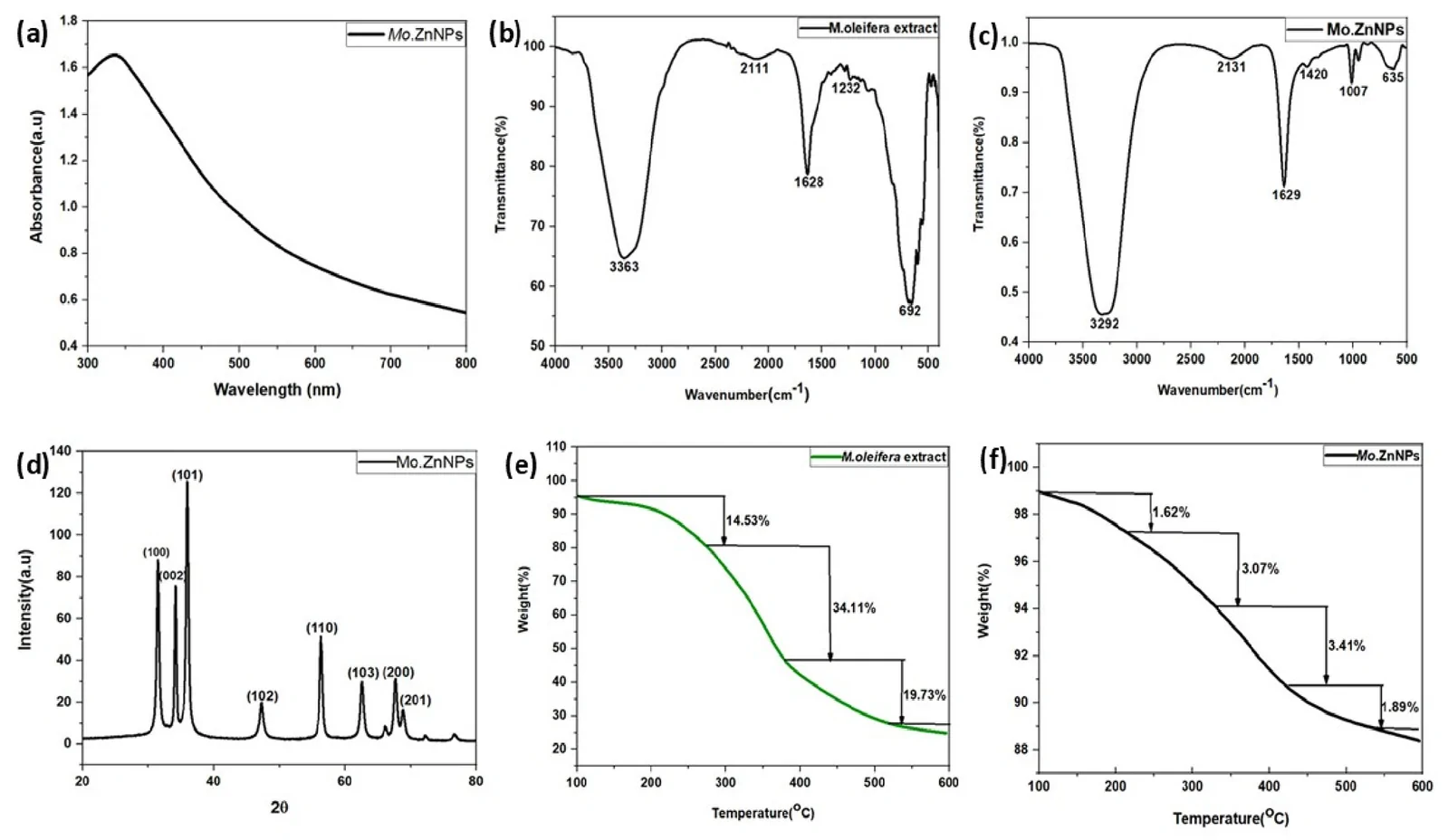
Characterization techniques of zinc nanoparticles. (**a**) The zinc nanoparticle synthesized from *M. oleifera* shows a maximum absorbance at 330 nm. (**b**) FTIR spectral analysis of *M. oleifera* leaf extracts. (**c**) FTIR spectral analysis of zinc nanoparticles synthesized from these leaf extracts. (**d**) XRD spectral analysis of zinc nanoparticles. (**e**,**f**) Thermogravimetric analysis of *M. oleifera* extracts and zinc nanoparticles.

Synthesis of ZnONPs using *M. oleifera* extracts was indicated by a change in color from white to golden and from yellowish to whitish in Zinc (Figure 1a). Zinc nanoparticles displayed peaks at 330 nm related to surface plasmon resonance of metal electrons and their excitation during nanoparticle synthesis [69,70]. This finding indicates that the optical properties of the zinc acetate solution changed due to the reduction of this salt to elemental zinc and further to zinc nanoparticles when exposed to bioactive compounds [71].

### 3.2. Fourier Transform Infrared Spectroscopy (FTIR)

FTIR measurements analyzed the *M. oleifera* extract biomolecules responsible for reducing Zn ions from the precursor salt and those that act as capping agents for ensuring NP stability. As shown in Figure 1b,c, FTIR spectral analysis was performed between 4000 and 500 cm^−1^ in the % transmittance (%T) mode. The resultant peaks included those of *Moringa oleifera* extracts (3343 and 1636 cm^−1^) and ZnONPs (3324, 2128, and 1638 cm^−1^). Their absence in the synthesized nanoparticles and a slight shift in peaks prove that these functional groups are involved in bio-reductive synthesis.

### 3.3. X-Ray Diffraction Studies

The synthesized ZnONPs were assessed using the XRD method. ZnONPs showed distinct peaks at 2ϴ°s of 69.1, 67.9, 63.1, 56.3, 47.4, 36.1, 34.5, and 31.7, which matched the (201), (112), (103), (110), (102), (101), (002), and (100) planes, respectively. The diffraction peaks matched well with those in the literature, confirming a wurtzite structure [46]. The data presented in Figure 1d indicates the crystalline nature of the nanoparticles.

### 3.4. Thermogravimetric Analysis (TGA)

To study the thermal stability of *Moringa oleifera* extracts and ZnONPs, thermogravimetry was conducted at higher temperatures from 100 to 600 °C. Figure 1e represents a % loss of 14.53% from 200 to 300 °C, indicating the disappearance of volatile compounds. Later on, during an increase from 300 to 400 °C, the % loss was 34.11%; at further heating up to 600 °C, it was about 19.73%, indicating that most of the volatile components were lost between 300 and 400 °C. ZnONPs exhibited a continuous weight loss at various thermal stages: 270, 380 and 490 °C, followed by a plateau. The initial stage loss occurred above 100 °C due to the volatiles formed during nanoparticle synthesis. The third peak at 490 °C could be assigned to the production of stable zinc oxide nanoparticles achieved by the decomposition of adsorbed surfactant molecules (Figure 1f).

### 3.5. FE-SEM and EDAX Analysis

**Figure 2:**
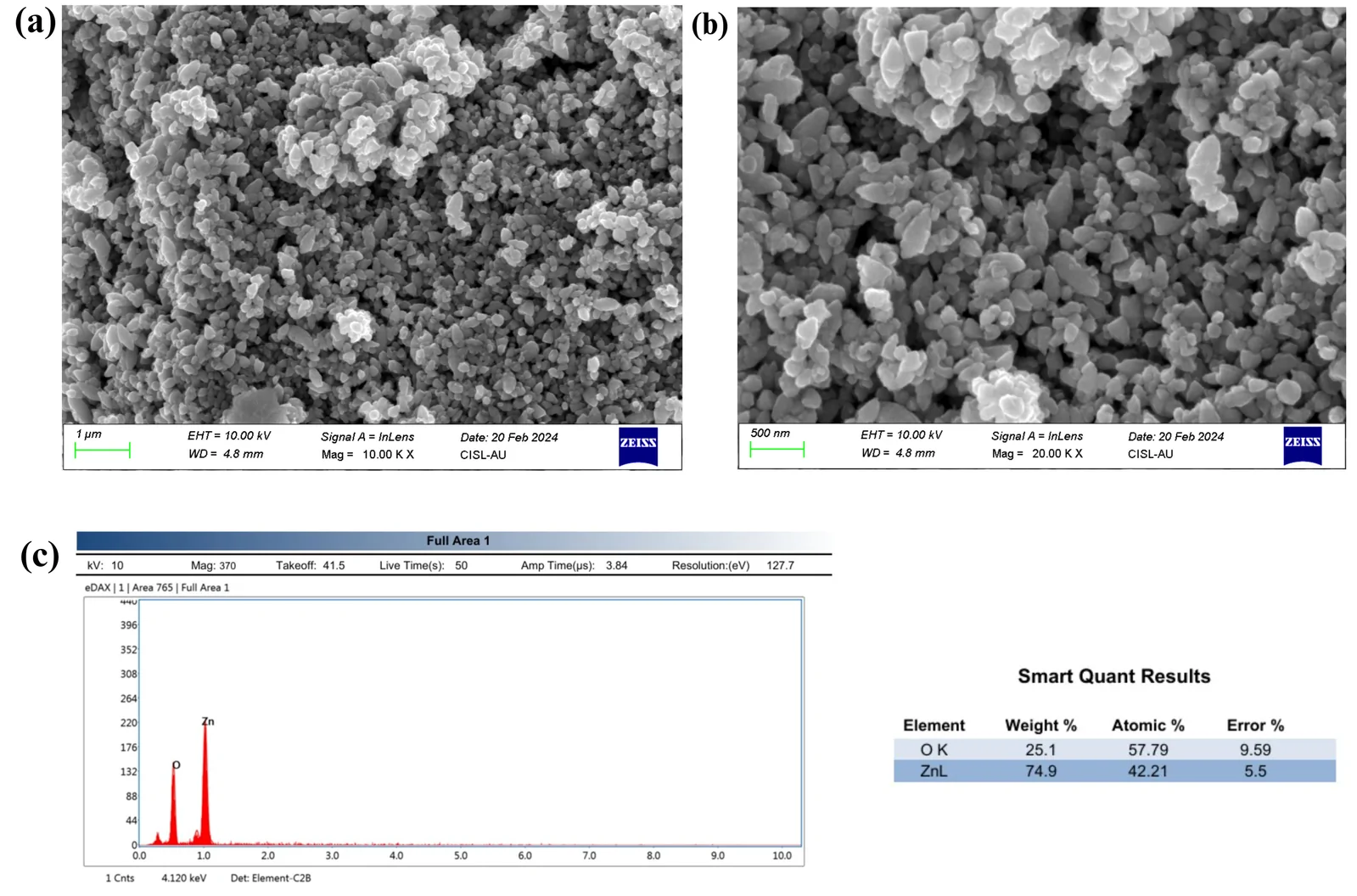
FESEM images of zinc nanoparticles synthesized from *M. oleifera* extracts showing a triangular shape (**a**,**b**). EDAX analysis indicates that the nanoparticles were in a pure form (**c**).

FE-SEM is an advanced technique utilized to view the external morphology of the synthesized zinc nanoparticles. They were triangular and obtained using extracts from the same plant, as presented in Figure 2a,b [72]. The presence of Zn was confirmed by EDAX spectral analysis, which indicated that the sample was in a pure form without any contamination by other elements (Figure 2c).

### 3.6. TEM

**Figure 3:**
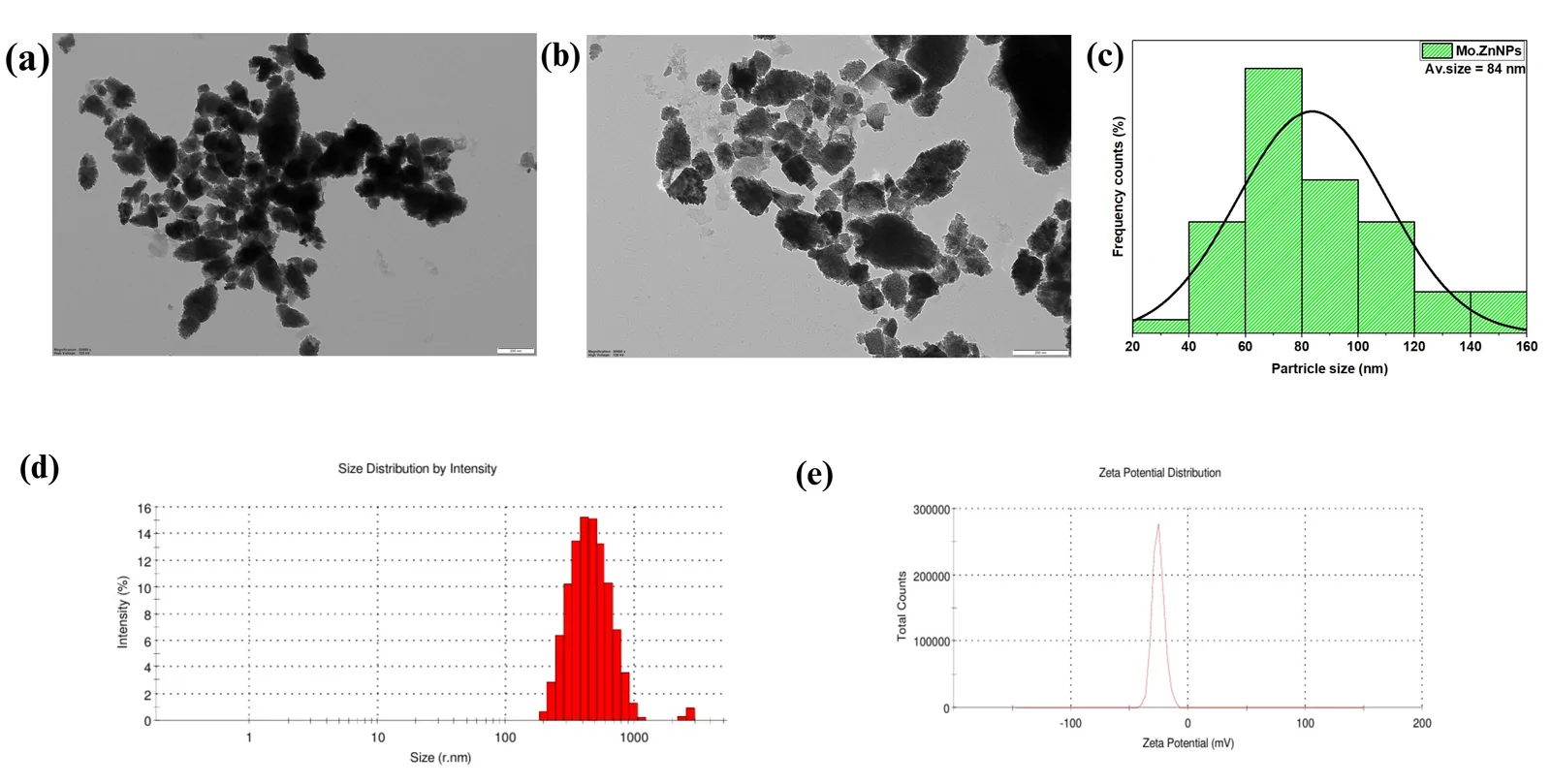
TEM images and particle size distribution histogram of ZnONPs (**a**–**c**). DLS (hydrodynamic size) and zeta potential analyses (**d**,**e**).

TEM is a well-known technique employed for imaging nanomaterials at atomic resolution. The images presented in Figure 3a–c indicate that the zinc nanoparticles synthesized had a triangular shape, and histogram analysis showed that the average size was 84 nm [73,74].

### 3.7. Zeta and DLS Analysis

The zeta potential and particle size distribution values of the synthesized nanoparticles were evaluated employing dynamic light scattering and a NanoZeta Sizer. DLS was used to estimate the average size of ZnONPs in solution, which was found to be 396 nm (Figure 3d). The larger size of particles in suspension suggested strong agglomeration behavior in colloidal solutions. To determine the surface charge and quantify the magnitude of charge that stabilized the nanoparticles in dispersion, the zeta potential was ascertained [75], which was −25.4 mV (Figure 3e).

### 3.8. Antioxidant Efficacy of ZnONPs

**Figure 4:**
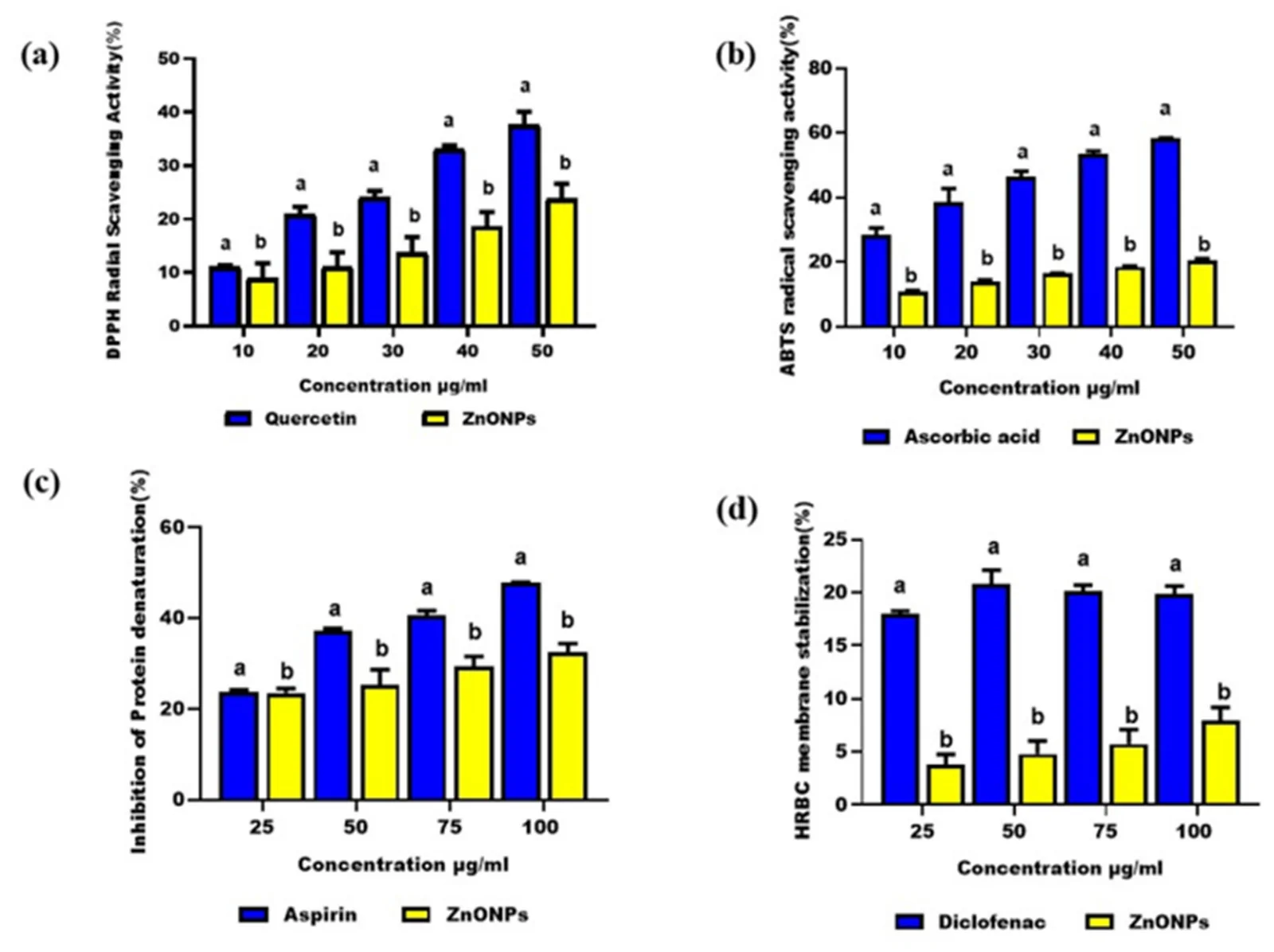
Antioxidant and anti-inflammatory properties of various concentrations of ZnONPs: (**a**) DPPH radical scavenging activity. (**b**) ABTS radical scavenging activity, (**c**) albumin denaturation, and (**d**) HRBC membrane stabilization. Statistical significance was determined by one-way ANOVA followed by Tukey’s multiple-comparison test. Different lowercase letters indicate significant differences among treatments (*p* < 0.05), while bars sharing the same letter are not significantly different. Values are presented as mean ± SD (n = 3).

The antioxidant effects of the zinc nanoparticles were evaluated using the 2,2-diphenyl-1-picrylhydrazyl radical (DPPH) assay; an increase was observed due to an enhancement in nanoparticle concentrations. DPPH is a well-known compound that can accept electrons in its free form. The reaction was detected through color change after adding ZnONP solutions. Figure 4a shows that Zinc nanoparticles demonstrated inhibition in a concentration-dependent manner. The percentages of DPPH scavenging activity were 9, 11, 13, 18, and 23 at NP concentrations of 10 to 50 μg/mL. The IC_50_ values for ZnONPs and Quercetin were 34.01 and 26.28 μg/mL, respectively. ABTS radical scavenging activity of ZnONPs is presented in Figure 4b. Zinc nanoparticles showed inhibition in a concentration-dependent manner, with % inhibitions of 10, 13, 16, 18, and 20 at concentrations of 10 to 50 μg/mL. IC_50_ values for ZnONPs and ascorbic acid were 26.16 and 25.23 μg/mL, respectively.

### 3.9. Anti-Inflammatory Efficacy of ZnONPs

Anti-inflammatory activities of ZnONPs were examined via protein denaturation assays and HRBC membrane stabilization methods. Figure 4c shows the % inhibition of egg albumin denaturation from 25 to 50 μg/mL, which was dose-dependent. IC_50_ values of ZnONPs and aspirin were 65.14 and 50.46 μg/mL, respectively. The % inhibition of protein denaturation for ZnONPs was 23, 25, 29, and 32 at 25, 50, 75, and 100 μg/mL, respectively.

HRBC membrane stabilization is one technique used to study anti-inflammatory activity. RBC membrane closely resembles the lysosomal membrane. Hypotonicity-induced stabilization was used to test ZnONPs. Figure 4d shows that the % inhibition of nanoparticles in a concentration-dependent manner was 3, 4, 5, and 8 at 25, 50, 75, and 100 μg/mL, respectively. IC_50_ values of ZnONPs and diclofenac were 69.86 and 35.52 μg/mL, respectively.

### 3.10. Antibiofilm Activities of ZnONPs

**Figure 5:**
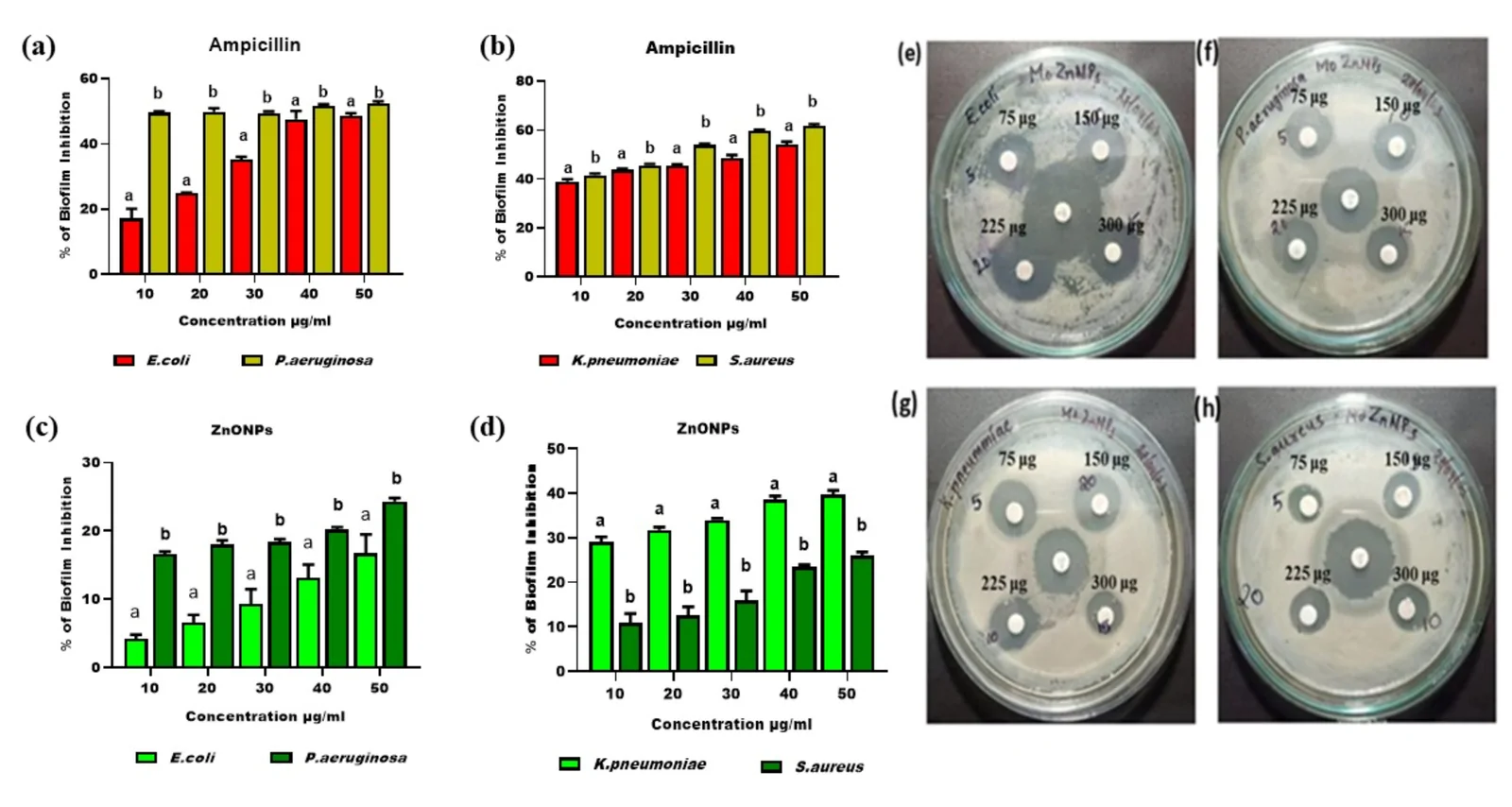
Antibiofilm activity of ZnONPs at different concentrations assessed utilizing disk diffusion assays. (**a**,**b**) show the % biofilm of the standard (ampicillin); (**c**,**d**) show the % biofilm of varying concentrations of ZnONPs. (**e**–**h**) Zones of inhibition related to MDR bacteria such as (**a**) *Escherichia coli*, (**b**) *Pseudomonas aeruginosa*, (**c**) *Klebsiella pneumoniae*, and (**d**) *Staphylococcus aureus*. Statistical significance was determined by one-way ANOVA followed by Tukey’s multiple-comparison test. Different lowercase letters indicate significant differences among treatments (*p* < 0.05), while bars sharing the same letter are not significantly different. Values are presented as mean ± SD (n = 3).

The antibiofilm activity of ZnONPs was evaluated via the microplate method. The nanoparticles inhibited MDR bacteria in a concentration-dependent manner. Each bacterial species was grown separately before the day of the experiment, inoculated into 96-well plates, and each was treated with ZnONPs at 10, 20, 30, 40, and 50 μg/mL. The % inhibition of the ZnONPs was 4, 6, 9, 13 and 16% (*E. coli*), 16, 18, 18.3, 20 and 24% (*P. aeruginosa*), 29, 31, 33, 38 and 39% (*K. pneumoniae*) and 10, 12, 15, 23 and 25% (*S. aureus*), as presented in Figure 5a–d.

## 4. ZnONPs Show Potent Activity Against MDR Bacteria

Antibacterial activities of ZnONPs were estimated using the disk diffusion method, which showed effective antibacterial activity against MDR bacteria (*Escherichia coli*, *Pseudomonas aeruginosa*, *Klebsiella pneumoniae*, and *Staphylococcus aureus*) isolated from wound infections. Ciprofloxacin (5 µg)-containing disks served as a standard control. The results suggested effective activity of ZnONPs against Gram-positive (*S. aureus*) and Gram-negative (*E. coli*, *K. pneumoniae*, and *P. aeruginosa*) MDR, as the zones of inhibition were directly proportional to the concentrations 75,150, 225, and 300 µg/mL (Figure 5e–h). ZnONPs induced a 6 mm zone of inhibition at 300 µg/mL against all bacteria except *S. aureus*.

## 5. Improvement of Agronomic Traits and Yield-Associated Characteristics Under ZnONP Application

Only the significant results regarding different growth and yield-related parameters under treatment with different levels of ZnONPs are presented.

### Seedling Growth

**Figure 6:**
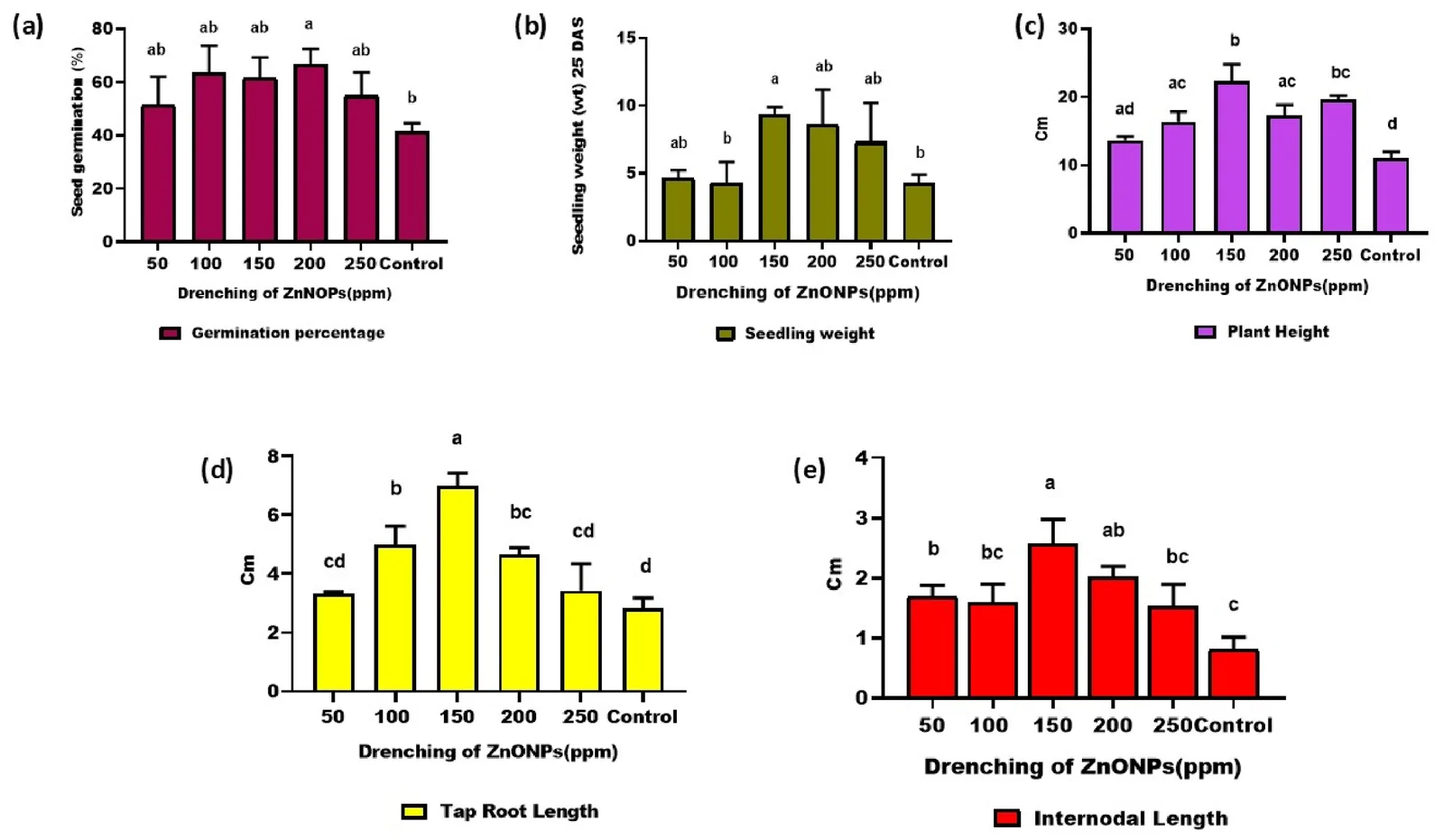
Effects of hydropriming and ZnONP drenching on various seedling growth characteristics of tomato, such as (**a**) Germination percentage, (**b**) Seedling weight, (**c**) Plant height, (**d**) taproot length, and (**e**) Internodal length. Statistical significance was determined by one-way ANOVA followed by Tukey’s multiple-comparison test. Different lowercase letters indicate significant differences among treatments (*p* < 0.05), while bars sharing the same letter are not significantly different. Values are presented as mean ± SD (n = 3).

Seed hydropriming and subsequent drenching with ZnONPs improved seedling characteristics at the nursery stage. The % germination was significantly positively correlated among treatments (Table 1). Hydropriming of the seeds with T_4_ resulted in a germination percentage of 66.67 ± 5.77, the highest among all other treatments with statistical significance, followed by T_3_ and T_2_; however, the impacts of these two treatments were insignificant, as depicted in Figure 6a. Seedling weight and plant height were determined 25 days after sowing. Among treatments with different levels of ZnONPs, the maximum seedling weight was recorded with T_3_ at 9.33 gm, followed by T_4_ at 8.67 gm, and T_5_ at 7.33 gm. All three treatments significantly enhanced seedling weights (*p* < 0.05) compared to the control, but the inter-treatment weights did not vary with statistical significance, as indicated in Table 1 and Figure 6b. The greatest plant height was recorded with T_3_ at 22.33 cm, followed by T_5_ at 19.67 cm, and T_4_ at 17.33 cm. The differences among T_3_, T_5_, and T_4_ T_5_ were statistically non-significant, as represented in Table 1 and Figure 6c. The tap root length of the plant determines seedling growth and lateral root production. The highest tap root length was observed in T_3_ at 7.00 cm, followed by T_1_ at 5.00 cm. The extended internodal length encouraged fruit set and ripening; elevated dosages of ZnONPs extended internodal length. T_3_ resulted in an internodal length of 2.56 cm, followed by T_4_ at 2.03 cm. The results for T_1_ and T_2_ were statistically insignificant. Overall, different ZnONP applications caused statistically significant variations compared to the control, ZnOSO_4_.

## 6. Morphometric Parameters

**Figure 7:**
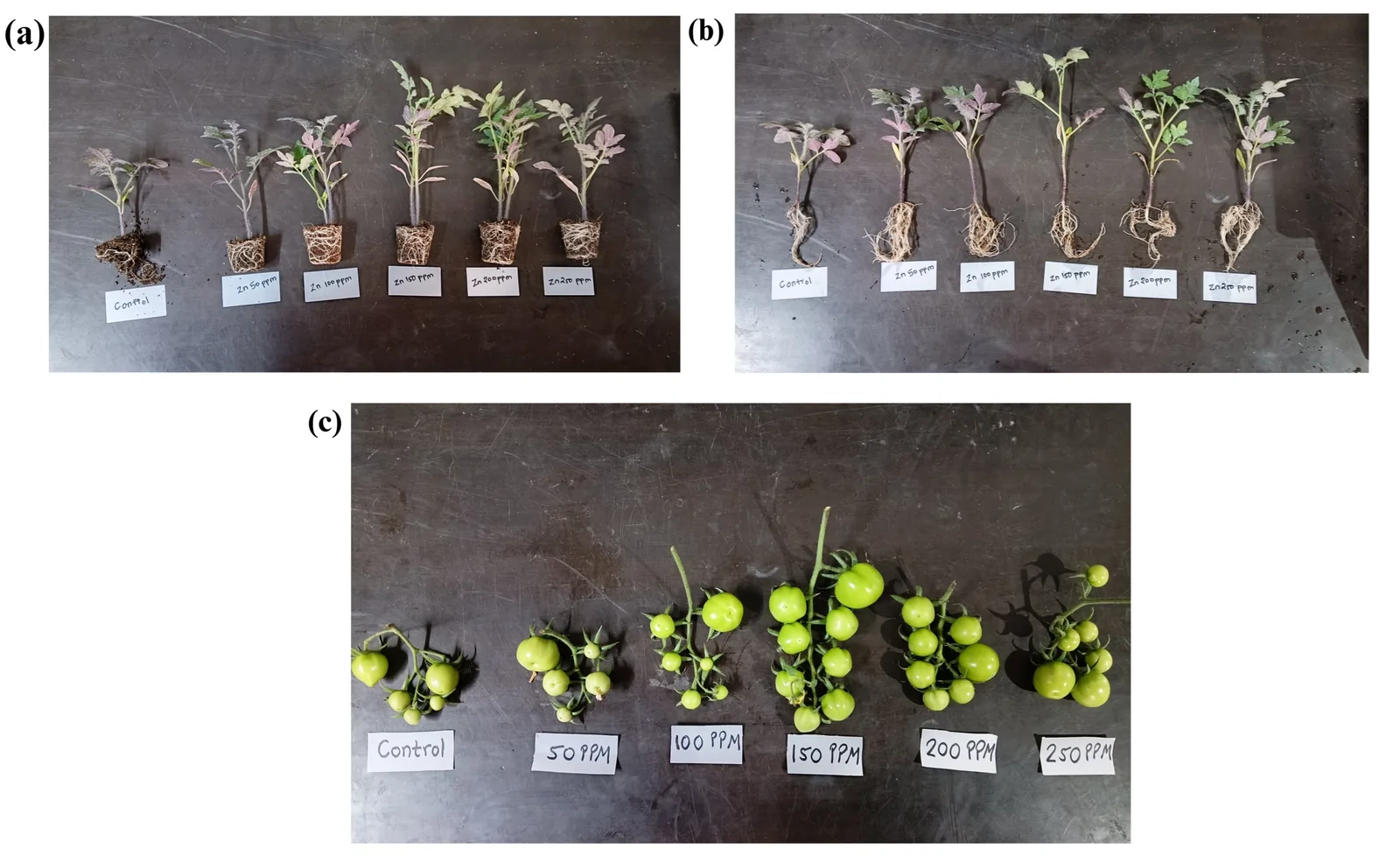
(**a**,**b**) Effect of hydropriming with ZnONPs on growth quality parameters of tomato seeds, (**c**) Impacts of foliar application of ZnONPs on tomato fruit yield per plant.

Foliar application of ZnONPs induced a great variation among morphometric parameters after transplanting from the nursery. Early flowering is an advantage; foliar application greatly altered the phytohormone levels, which induced early flowering and also prominently impacted flower development. Early flowering emergence was observed under T_3_ at 34.33 DAT, followed by T_4_ at 38.00. These differences among the two treatments were statistically significant (*p* < 0.05); the highest days to flowering was with the control at 52.33 DAT. The significance among the treatments is represented in Table 2 and Figure 7a. Leaf area was remarkably increased under foliar application in T_4_ at 13.56 cm, followed by T_3_ at 13.19 cm (statistically significant), and T_4_ at 13.00 cm (statistically insignificant) compared to T_4_ and T_3_, as represented in Table 2 and Figure 7b. Chlorophyll content directly impacts photosynthetic ability; ZnONPs positively enhanced the leaf chlorophyll content. The photosynthetic ability was recorded with a SPAD meter. The highest SPAD values were recorded in T_3_ at 72.00, followed by T_4_ at 72.33; the variations were statistically significant among treatments, but insignificant with each other at *p* < 0.05. T_5_ was at 65.67; the lowest recorded was with T_1_ at 52.00 and the control at 48.33. However, the chlorophyll content increased at T_3_ and T_4_ (Figure 7c). Maximum fruit count was observed in T_3_ at 64.00, followed by T_4_ at 56.00, T_5_ at 52.00, T_2_ at 43.00, and the lowest in T_1_ at 29.33, compared to the control at 33.00. The highest number of fruits per cluster was in T_3_ at 11.00, T_4_ at 9.00, T_2_ and T_5_ at 7.00, T_1_ at 5.33, and the least in the control at 5.00. Statistical significance is presented in Table 2 and Figure 7.

## 7. Yield and Quality-Related Parameters

**Figure 8:**
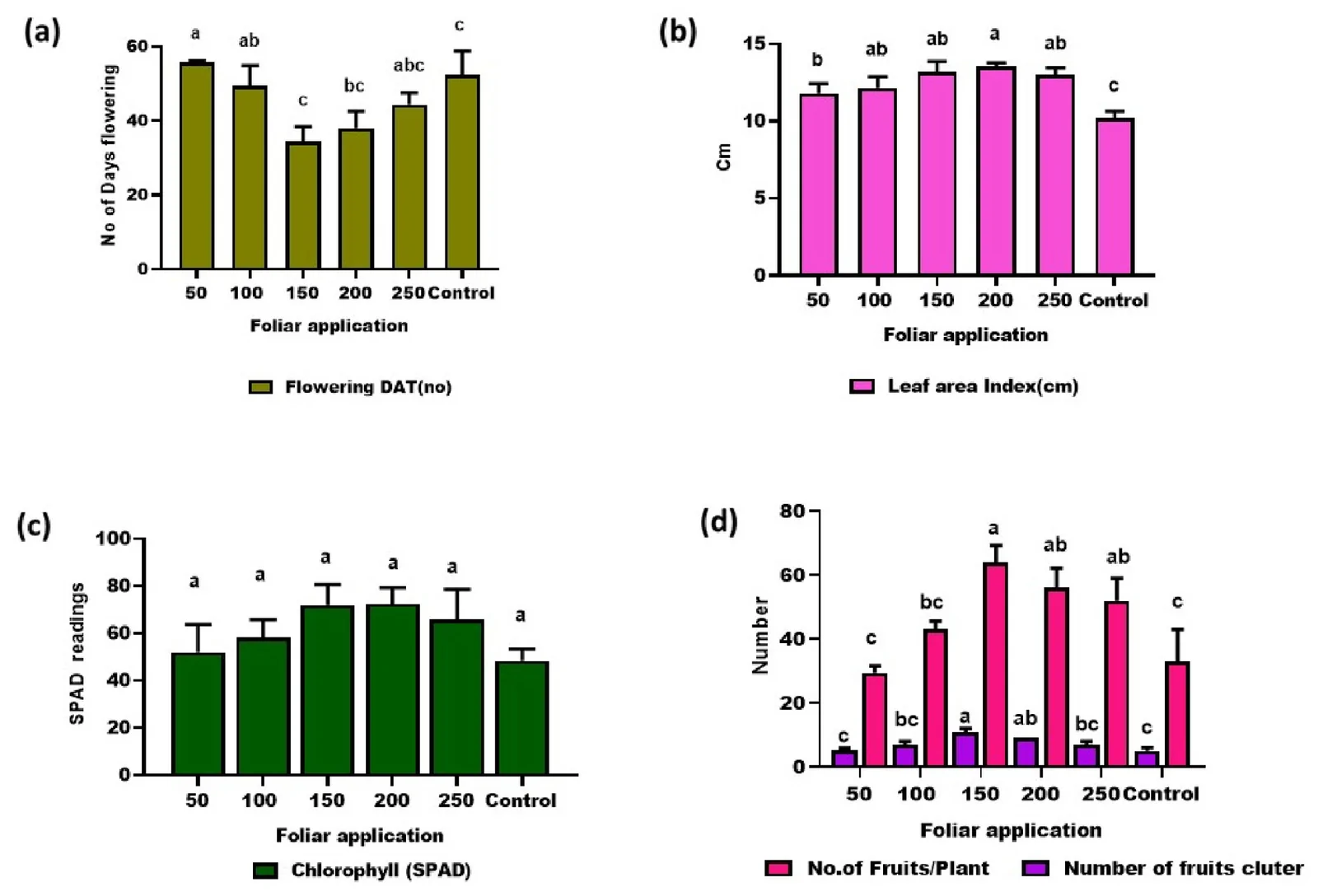
Influence of foliar application of ZnONPs on different morphometric characteristics such as (**a**) Flowering days after transplanting, (**b**) Leaf area (cm), (**c**) Chlorophyll SPAD, (**d**) Number of fruits per plant or Number of fruits per cluster. Statistical significance was determined by one-way ANOVA followed by Tukey’s multiple-comparison test. Different lowercase letters indicate significant differences among treatments (*p* < 0.05), while bars sharing the same letter are not significantly different. Values are presented as mean ± SD (n = 3).

Fruit diameter varied insignificantly among treatments, with the greatest fruit diameter seen in T_2_ (4.70 ± 0.85), T_3_ (4.63 ± 0.06), T_4_ (4.23 ± 0.57), and T_5_ (4.17 ± 0.81); the lowest diameter was observed in the Control (3.53 ± 0.87) and T_1_ (3.83 ± 0.60). Total yield was recorded from transplanting to final harvesting under foliar application with ZnONPs; the highest weight was observed in T_3_ at 4102.63 g, followed by T_4_ at 3994.97 g, T_5_ at 3786.23 g, T_2_ at 3547.23 g, T_1_ at 3071.63 g, and the least in the control at 2847.30 g, the results of all treatments were markedly superior over the control. Marketable yield of tomatoes represents the net clean quantity after sorting and grading. The highest marketable yield was recorded in T_3_ at 3984.30 g, followed by T_4_ at 3884.30 g, T_5_ at 3625.57 g, T_2_ at 3413.57 g, T_1_ at 2954.30 g, and the least in the control at 2723.97 g. TSS represents the available sugars, generally fructose, for processing; the greatest TSS Brix values were observed in T_3_ at 9.73, T_4_ at 7.90, and T_2_ at 6.03, while the lowest were in T_5_ at 5.23 and T_1_ at 5.00, and the lowest was in the control at 4.20. Shelf life is an important qualitative parameter; that of tomatoes was calculated under optimum room temperature starting from the date of harvest. The highest shelf life was observed in T_3_ at 15.33, followed by T_4_ at 14.33, T_5_ at 12.67, T_2_ at 11.33, T_1_ at 9.33; the lowest was recorded in the control at 7.33. The statistical significance of the data on yield-attributing characters is represented in Table 3 and Figure 8.

## 8. Management of Late Blight in Tomatoes

**Figure 9:**
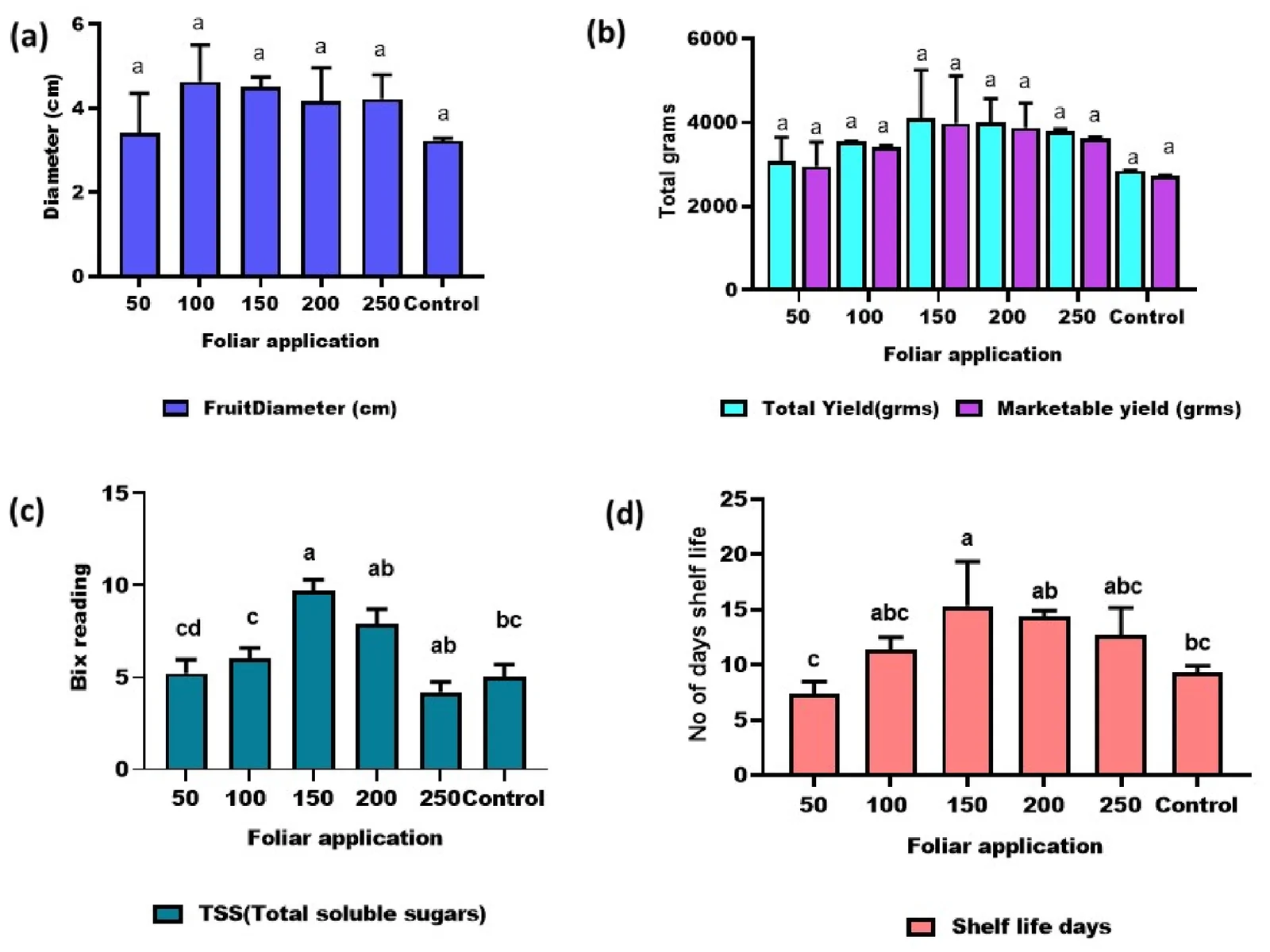
Impacts of foliar application of ZnONPs on different morphometric characteristics such as (**a**) Fruit diameter, (**b**) Total yield and Marketable yield, (**c**) TSS, and (**d**) Shelf life of Tomatoes. Statistical significance was determined by one-way ANOVA followed by Tukey’s multiple-comparison test. Different lowercase letters indicate significant differences among treatments (*p* < 0.05), while bars sharing the same letter are not significantly different. Values are presented as mean ± SD (n = 3).

**Figure 10:**
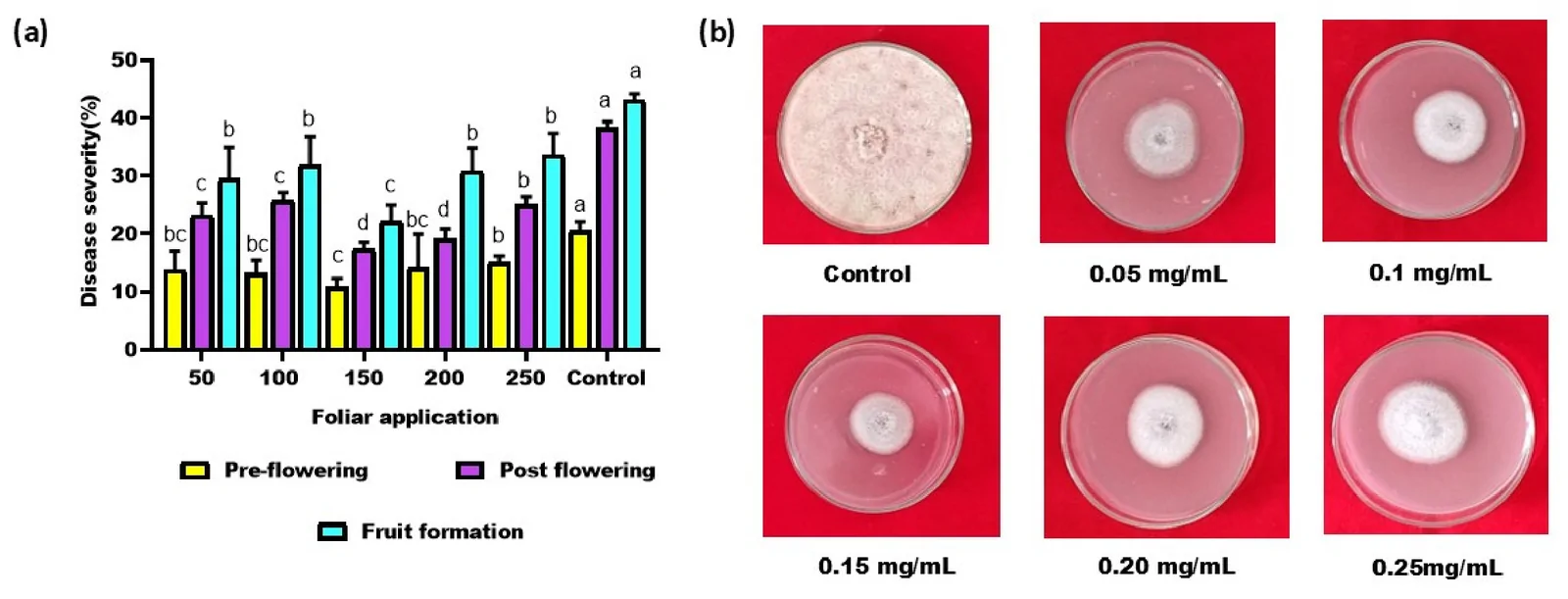
Effect of foliar application of different concentrations of ZnONPs on late blight disease severity (**a**) in vivo and (**b**) in vitro antifungal efficacy of ZnONPs against the late blight pathogen (*Phytophthora infestans*). Statistical significance was determined by one-way ANOVA followed by Tukey’s multiple-comparison test. Different lowercase letters indicate significant differences among treatments (*p* < 0.05), while bars sharing the same letter are not significantly different. Values are presented as mean ± SD (n = 3).

Late blight infection is a major biotic stress observed during the crop cycle of tomatoes under greenhouse conditions (Figure 9). Effective disease management strategies are crucial; ZnONPs were effective against late blight. Blight infection stage was recorded at three phenological stages before flowering. The lowest infection was observed in T_3_ at 10.82, followed by T_2_ at 13.18, T_1_ at 13.81, T_4_ at 14.21, T_5_ at 15.18 and the highest in the control at 20.61. Statistically non-significant variations were observed between T_1_ and T_2_. However, all the treatments induced statistically significant differences in disease severity, *p* < 0.005 at the pre-flowering stage. Post-flowering blight infection increased under favorable climatic conditions under foliar applications of Zn-OPs: in T_3_ at 17.42, followed by T_2_ at 19.09, T_1_ at 23.33, T_4_ at 25.08, T_5_ at 25.08, and the lowest in the control at 38.31. The most severe infection during the fruiting stage under foliar application was observed in the control at 48.96, but disease reduction varied significantly under application of ZnONPs at different levels; the lowest was in T_3_ at 20.02, followed by T_2_ at 27.57, T_4_ at 32.33, T_5_ at 33.62, and T_1_ at 40.94; in all treatments, the data differed statistically significantly compared to the control. In vitro evaluation of antifungal efficacy against the late blight pathogen under different concentrations of ZnONPs revealed the lowest fungal growth (mm) in T_3_ or 0.15 mg/mL at 19.50, followed by T_2_ at 28.60%, T_4_ at 30.80%, T_5_ at 34.17%, and T_1_ at 50.97%; the highest was in the control at 92.90 ± 10.86. The in vitro and in vivo evaluations of the antifungal activities of ZnONPs and their statistically significant variations are represented in Table 4 and Figure 10a,b.

Mean values of foliar, in vitro, and in vivo application of ZnONPs on different parameters of tomatoes within a column that include the same letters were not statistically different at the 5% and 1% levels ascertained using Tukey’s HSD test. CD = Critical difference, CV = Coefficient of variation, T1 = 50 ppm, T2 = 100 ppm ZnONP nutrient, T3 = 150 ppm Zn nutrient, T4 = 200 ppm ZnONPs nutrient, T5 = 250 ppm ZnO nanoparticle, T6 = ZnO nanoparticle, Note: ns-not significant at *p* > 0.05, * significant at *p* > 0.05 and ** significant at *p* > 0.01; standard error (n = 5); (ANOVA).

## 9. Discussion

Nanoparticles exhibit many advantages, such as stability, large surface area, and various applications in medicine, drug delivery, and disease diagnostics. Green synthesis approaches have started attaining more attention for producing metal nanoparticles because they are more reliable, cost-effective, fast, and environmentally friendly than other methods [76]. The present study describes the synthesis of zinc nanoparticles from *M. oleifera* leaf extracts. An absorbance peak at 340 nm indicated the formation of zinc nanoparticles [77]. XRD peaks show a pattern similar to several reports, showing the crystalline and wurtzite structure of ZnONPs [77,78,79]. FTIR spectrum analysis identified numerous functional groups; similar nanoparticles were reported earlier [80]. The 1638 and 2128 cm^−1^ bands in zinc nanoparticles indicate the presence of carboxylic acid, (C–N) stretching, and aliphatic amines [80,81,82,83]. FE-SEM and TEM analysis revealed the surface morphology to be triangular [72]. TEM analysis demonstrated the average size of the nanoparticles to be 84 nm. EDAX analysis indicated the elemental composition as oxygen and zinc. DLS confirmed the size and distribution of the nanoparticles, showing a size of 96 nm. Surface charge confirmed by zeta potential analysis revealed a value of 25.4 mV for the zinc nanoparticles [84]. TGA suggested complete decomposition of the ZnONPs at 600 °C due to the volatile nature of bioactive phytochemicals [85].

Antioxidant activity of ZnONPs was determined using DPPH and ABTS radical scavenging assays. The excellent antioxidant activity of the synthesized nanoparticles was due to the presence of secondary metabolites such as saponins, phenols, amino acids, alkaloids, flavonoids, cardiac glycosides, and coumarins [86]]. Secondary metabolites may play a prominent role in stabilizing lipid oxidation, linked to antioxidant activity [87]. Numerous nanotechnology studies have used different plant extracts. However, no report is available on zinc nanoparticles synthesized using *M. oleifera* aqueous leaf extracts. The ZnONPs showed maximal inhibition of protein denaturation, 32% at 100 μg/mL. ZnONPs demonstrated promising anti-inflammatory activities and efficient stabilization of the cell membranes, suggesting potential anti-inflammatory drugs. Using extracts from *Kalanchoe pinnata* and *Euphorbia retusa* for green synthesis was better at stopping the production of cytokines and reducing inflammation by activating caspase-8 proteases. The phytochemical-loaded zinc nanoparticles might contribute to improving anti-inflammatory activities [88,89]. Biosynthesized zinc nanoparticles exhibit antimicrobial mechanisms by decreasing the integrity of bacterial cell walls and affecting cell membranes, changing bacterial cell function, inhibiting electron transport, and finally damaging the genetic material. This study highlighted activity against multidrug-resistant clinical isolates, such as *E*. *coli*, *P. aeruginosa*, *K*. *pneumoniae*, and *S. aureus.* For the first time, we used varied concentrations of ZnONPs and measured the zones of inhibition against these four MDR bacteria. Antibacterial efficacy was examined against *S. aureus and E. coli,* and only these two were inhibited [90]. ZnONPs exhibited relatively small zones of inhibition against the tested bacterial isolates. In all cases, the zones of inhibition were considerably smaller than those produced by ciprofloxacin, indicating that ZnONPs possessed only moderate antibacterial activity under the experimental conditions applied.

Biofilm production causes resistance to numerous antibacterial agents; thus, biofilm inhibition is essential for preventing infections and other disorders [91]. The antibiofilm ability of ZnONPs was assessed against the four MDR bacteria [63]. ZnONPs exhibited a moderate % inhibition against all MDR species, which penetrate cells and bind to DNA. *S. aureus* showed the greatest resistance to all concentrations of ZnONPs, which might be due to bacteria targeting the weak links within colloidal dispersions of nanoparticles. The aggregate stability was minimal due to the environmental and physical conditions of the nanoparticle solutions, and repeated exposure of *S. aureus* to ZnONPs also lowered the inhibition rate [92,93].

Zinc nanoparticles exhibited moderate anti-inflammatory, antibacterial, and antibiofilm activities. To test the efficacy of these zinc oxide nanoparticles on tomato plants, we conducted greenhouse trials with different concentrations of nanoparticles. Hydropriming tomato seeds with ZnONPs at the nursery stage induced great variations in seedling parameters. Healthy seedlings are most important for good crop establishment and achieving lucrative yields. Zn-based seed priming is a prominent technique for advancing seedling growth in different crops like maize, wheat, and soybean under varied abiotic stresses [94,95,96]. In vitro generation of *Arabidopsis thaliana* under Zn levels ranging from 1 to 250 µm showed a remarkable correlation between Zn levels and root development in *Arabidopsis thaliana* [97]. The maximum germination percentage of tomato seeds was recorded with T4 at 66.67%. These results correlate with previous findings; ZnONPs at different concentrations showed varied results, as concentrations from 700 to 2000 ppm recorded germination percentages from 70% to 85%. But interestingly, ZnONPs at 400 ppm enhanced germination percentage to above 90%, with 100 to 200 ppm enhancing it between 60% and 70% [98].

Similarly, a distinct influence on plant height was observed, with the maximum at 150 ppm ZnONPs at 22.33 cm, 20 days after sowing; foliar and drenching with ZnONPs improved growth parameters, significantly increasing plant height; it also influenced seedling parameters: leaves, stem, and dry weight of plants compared to the control [99]. Foliar application of pinto bean ZnONPs induced a maximum height of 90 cm compared to the control due to extended internodal length [100]; similarly in the present report, the highest internodal length was with ZnONPs at 150, 200, and 250 ppm in that order. Research findings showed that Nano Zinc application as a foliar spray or drenching enhanced plant height in different crops at desirable concentrations [101,102]. Fruit length and diameter indicate the quality of tomatoes and ultimately the yield of plants; the diameter of tomatoes ranged from 3.58 in the control to 4.70 under 100 ppm. However, the treatments produced results that were statistically similar to those under foliar application of ZnONPs at 25, 50, 75, and 100 pm with leaf areas of 3.02, 3.55, 4.05, 4.16, and 4.33 cm^2^. Similarly, the highest number of fruits was at 100 ppm, at approximately 52.75/plants [103], which correlates with the present findings, suggesting that the fruit number increased with dosages but in a similar range.

Foliar application of ZnONPs exerted a detrimental effect on yield-contributing factors and various growth parameters at different morphometric stages. These traits are crucial to evaluating the impacts and efficacy of foliar-applied ZnONPs. Flowering is the most important trait in determining the effect of ZnONPs; hence, after the nursery stage, the plants were transplanted to the generative phase, where the productivity targets were achieved. Early flowering initiation post-transplanting is the most crucial impact of foliar ZnONP application at 150 and 200 ppm, which was 34.33 and 38.00 DAT, respectively. Compared to others, the differences between these two treatments were statistically insignificant. Similar results were reported in *Eustoma grandiflorum,* which was due to an increase in auxin concentrations [104]. Maximum flowering in plants was observed under foliar spray with nano Zinc at 250 ppm, with the maximum fruit setting and flowering at higher concentrations [105].

Leaf area has a greater role in plant photosynthetic ability. Plants with higher chlorophyll content generate more amounts of starch, with zinc at very low concentrations stimulating chlorophyll biosynthesis in *Phyllostachys pubescens* [106]. Foliar application of ZnONPs at 50, 100, and 200 ppm increased the leaf area of tomato plants by 13.8, 13.0, and 12.8 cm, respectively. Enhanced dosages of nanoparticles exert detrimental effects on leaf area [65], as was seen with the present research findings. Previous research on ZnONPs under pot experiments increased the chlorophyll A and B contents of the leaves by 24.6 and 10% in wheat at 0.12 g pot^–1^ [107]. Foliar application of nano zinc at 10, 20, and 30, or of Fe, Zn, and Mn nanochelates increased the chlorophyll leaf area index in soybean under drought conditions; a combination foliar spray of nano Fe + Zn induced the most significant influence among treatments [108]. The present findings suggest that the SPAD reading, which helps determine the chlorophyll content of leaves, was highest under foliar application, which positively enhanced plant growth. Foliar application of Nanozinc at 100 ppm recorded the maximum chlorophyll content (51.6 SPAD) in leaves [109], similar to the present investigation.

Number of fruits per cluster and total number of fruits per plant significantly increased at 150, 200, and 250 ppm. These results were surprisingly different compared to previous research findings [110]. Foliar application of ZnONPs at 50, 100, 150 ppm, along with RDF, showed the best results in increasing yield and other growth parameters among solanaceous vegetables, capsicum, and tomatoes. The possible reason for varied results might be the reducing agent used in the synthesis of ZnONPs with different botanical extracts, as they have varied phytochemical properties with distinct impacts on different crops. Another study found that the foliar application of ZnONPs at 250 ppm improved the quality parameters of tomato crop [111].

### 9.1. Yield Parameters

Yield parameters such as fruit size and yield per plant were greatly influenced by the foliar application of ZnONPs at different concentrations; the dosage of 150 ppm with RDF recorded the maximum total tomato yield of 4102.63 gm/plant and marketable yield of 3984.30 gm/plant, while at 200 ppm, they were 3994.97 and 3625.57 gm/plant, respectively. However, all the treatments caused a significant increase in tomato crop yield compared to the control, with only 1.5 g/L of ZnSO4 + RDF. Present experimental findings can be correlated with previous research findings, where nanoparticles as foliar applications with different RDFs with half mineral fertilization at the recommended dose showed varied results. At higher dosages, the yield was reduced. However, adding 50% ZnONPs to the RDFs of mineral fertilizer increased yield in tomato plants [112]. The present yield results were strongly supported by previous findings where application of ZnONPs increased the yield of tomatoes [113]. Similarly, the application of phytosynthesized ZnONPs at 25, 50, 100 ppm increased the yield of cherry tomatoes compared to the control, besides showing good postharvest quality [114].

Total soluble sugars generally represent the amount of sugars present in the tomato during the ripening stage of fruit; generally, for medium-sized tomatoes it is between 5 and 7%, while cherry tomatoes have 9–15% [115]. Maximum TSS in tomatoes was recorded under foliar spray of B, Zn, and Cu at 250 ppm [116]. Foliar spray of nano zinc at 100 ppm increased the TSS of tomatoes to 7.58 Brix values [104]. Chemically synthesized ZnONPs enhanced Brix readings from 4.0 to 7.2 as the dosage of ZnONPs increased from 50 to 2500 ppm; foliar application of ZnONPs at 100 ppm recorded 9.05 Brix in tomato varieties, but in main plot varieties without any application, the Brix was 8.05. Thus, the Brix value is totally dependent on the genetic characteristics of a variety; however, plant nutrition might bring incremental changes in quality [117]. The present research recorded the highest Brix value of 9.73 at 150 ppm. With an increase up to 200 ppm, the Brix values declined to 7.90 and 6.03. The highest shelf life was observed at 150 PPM, and the lowest was in the control. The higher antioxidant and flavonoid levels in the tomatoes increased their shelf life [118,119]. Foliar application of nano-Zinc at 150 and 250 mg/L reduced the MDA content by 25% and 30%, respectively, compared to the control, which most highly influenced the growth and qualitative parameters of tomato [120]. Zinc plays a crucial role in carbohydrate metabolism by activating specific enzymes such as carbonic anhydrase, fructose-1, 6-bisphosphate, and aldolase [6]. Nano-ZnO application directly enhanced sugar contents. Nano-ZnO increased the concentration of soluble sugars in *Rosmarinus* significantly [121].

### 9.2. Effect of ZnONPs on Host–Pathogen Interaction and Microbial Efficacy

Late blight infection is the most important biotic stress that affects crop quality under cooler climatic conditions. In the present research, we studied the effects of ZnONPs on late blight infection. The foliar application of Nanozinc at 150 ppm remarkably reduced blight by 10.82% at pre-flowering, followed by 100 ppm, 13.18% at post-flowering stages, but disease severity was proportionately increased as the concentration of ZnONPs increased. Overall, foliar application of ZnONPs after fruit set resulted in the least disease severity observed with foliar application of 150 ppm, compared to other treatments. In vitro evaluation to test the efficacy of ZnONPs against *Phytophthora infestans* showed the lowest mycelial growth of 19.50 mm at 0.15 mg/mL with agar-doped concentrations, followed by 28.60 mm at 0.10 mg/mL, and 30.80 mm at 0.20 mg/mL. Very few studies have estimated the efficacy of metal nanoparticles with antifungal and antibacterial properties in agriculture to date. ZnO NPs at 70 nm and concentrations greater than 3 mmol l^−1^ inhibited the growth of *B. cinerea* and *P. expansum* [122,123,124]. Chemically synthesized CuO and ZnO nanoparticles reduced the radial growth of *Phytopthora infestans.* In vitro studies showed maximum mycelial inhibition at 20 mg/L to 30 mg/L ZnONPs. In vivo evaluations at 100 mg/L of ZnONPs and CuONPs showed a maximum efficacy of 70.64 [10].

## 10. Conclusions

The results and discussion indicated that ZnONPs positively affected the growth and development of tomatoes and also contributed to effective outcomes through the foliar application of ZnONPs. Hydropriming of tomato seeds at a concentration of 200 ppm showed the best results in seed germination; however, the 150 ppm ZnONPs proved to be the best among the foliar applications in improving the tomato morphometric and yield-attributing characteristics, where the highest marketable yield with the longest shelf life was observed among the different treatments and management of the blight infection under both in vitro and in vivo conditions. Therefore, foliar spray of ZnONPs at 150 ppm can be recommended for good tomato production under greenhouse conditions.

## Figures and Tables

**Table 1:** Effect of seed hydropriming and drenching with ZnONPs on seedling growth and quality parameters. Letters which are indicating same are referred as a stastically significant.

Treatments	Germination %	Plant Weight (g)	Plant Height (cm)	Tap Root Length (cm)	Internodal Length (cm)
T1	51.67 ± 10.41 ^bc^	4.67 ± 0.58 ^b^	13.67 ± 0.58 ^de^	3.33 ± 0.06 ^c^	1.69 ± 0.18 ^bc^
T2	63.33 ± 10.41 ^ab^	4.33 ± 1.53 ^b^	16.33 ± 1.53 ^cd^	5.00 ± 0.62 ^b^	1.59 ± 0.30 ^bc^
T3	61.67 ± 7.64 ^ab^	9.33 ± 0.58 ^a^	22.33 ± 2.52 ^a^	7.00 ± 0.44 ^a^	2.56 ± 0.41 ^a^
T4	66.67 ± 5.77 ^a^	8.67 ± 2.52 ^a^	17.33 ± 1.53 ^bc^	4.67 ± 0.23 ^b^	2.03 ± 0.16 ^b^
T5	55.00 ± 8.66 ^abc^	7.33 ± 2.89 ^a^	19.67 ± 0.58 ^ab^	3.43 ± 0.91 ^c^	1.53 ± 0.36 ^c^
T6	41.67 ± 2.89 ^c^	4.33 ± 0.58 ^b^	11.00 ± 1.00 ^e^	2.83 ± 0.35 ^c^	0.81 ± 0.20 ^d^
CD	13.8	2.17	2.85	0.95	0.45
MSE	57.50	1.42	2.46	0.27	0.06
SE(m)	4.38	0.69	0.90	0.30	0.14
SE(d)	6.19	0.97	1.28	0.43	0.2
CV(%)	13.38	18.51	9.37	11.95	14.38

**Table 2:** Effect of foliar application of ZnONPs on various morphometric characteristics of tomato fruits. Letters which are indicating same are referred as a stastically significant. Asterisks indicate significant at 0.05.

Treatments	Flowering DAT	Leaf Area	Chlorophyll SPAD	Number of Fruits Clusters	Number of Fruits per Plant
T1	55.67 ± 0.58 ^a^	11.80 ± 0.63 ^c^	52.00 ± 11.79 ^bc^	5.33 ± 0.58 ^d^	29.33 ± 2.31 ^e^
T2	49.33 ± 5.51 ^bc^	12.15 ± 0.73 ^bc^	58.00 ± 7.81 ^abc^	7.00 ± 1.00 ^c^	43.00 ± 2.65 ^cd^
T3	34.33 ± 4.04 ^d^	13.19 ± 0.68 ^a^	72.00 ± 8.72 ^a^	11.00 ± 1.00 ^a^	64.00 ± 5.29 ^a^
T4	38.00 ± 4.58 ^d^	13.56 ± 0.23 ^a^	72.33 ± 7.02 ^a^	9.00 ± 0.00 ^b^	56.00 ± 6.08 ^ab^
T5	44.33 ± 3.21 ^c^	13.00 ± 0.48 ^ab^	65.67 ± 12.90 ^ab^	7.00 ± 1.00 ^c^	52.00 ± 7.00 ^bc^
T6	52.33 ± 6.43 ^ab^	10.23 ± 0.43 ^d^	48.33 ± 5.03 ^c^	5.00 ± 1.00 ^d^	33.00 ± 10.00 ^de^
CD	5.67	14.18 **	3.67 *	1.62	10.77
MSE	9.70	0.00	0.04	0.79	35.06
SE(m)	1.80	1.02	16.78	0.51	3.42
SE(d)	2.54	0.31	85.06	0.73	4.83
CV (%)	6.82	0.32	5.32	12.02	12.81

**Table 3:** Effect of foliar application of ZnONPs on the fruit quality parameters. Letters which are indicating same are referred as a stastically significant.

Treatments	Fruit Diameter (cm)	Total Yield	Marketable Yield	TSS	Shelf Life
T1	3.43 ± 0.93	3071.63 ± 574.72 ^bc^	2954.30 ± 580.61 ^bc^	5.00 ± 0.70 ^d^	9.33 ± 0.58 ^cd^
T2	4.64 ± 0.87	3547.23 ± 3.49 ^abc^	3413.57 ± 32.27 ^abc^	6.03 ± 0.57 ^c^	11.33 ± 1.15 ^bc^
T3	4.53 ± 0.22	4102.63 ± 1149.76 ^a^	3984.30 ± 1131.31 ^a^	9.73 ± 0.59 ^a^	15.33 ± 4.04 ^a^
T4	4.17 ± 0.81	3994.97 ± 574.15 ^a^	3884.30 ± 577.26 ^a^	7.90 ± 0.82 ^b^	14.33 ± 0.58 ^ab^
T5	4.23 ± 0.57	3786.23 ± 55.64 ^ab^	3625.57 ± 33.69 ^ab^	5.23 ± 0.74 ^d^	12.67 ± 2.52 ^abc^
T6	3.23 ± 0.06	2847.30 ± 6.78 ^c^	2723.97 ± 13.70 ^c^	4.20 ± 0.56 ^e^	7.33 ± 1.15 ^d^
CD	2.23 ^NS^	841.23	846.53	0.75	3.78
MSE	0.13	349.72	597.55	0.17	4.32
SE(m)	-	266.97	268.65	0.24	1.20
SE(d)	0.45	377.55	379.93	0.34	1.7
CV (%)	0.39	12.99	13.56	6.47	17.74

**Table 4:** Efficacy of ZnONPs against disease severity in tomato plants at pre-flowering, post-flowering, and fruit formation stages, assessed via in vitro evaluation of mycelial growth inhibition. Letters which are indicating same are referred as a stastically significant.

Treatments	Pre Flowering	Post Flowering	Fruit Formation	In Vitro
T1	13.81 ± 3.26 ^bc^	23.33 ± 1.99 ^c^	31.61 ± 3.77 ^bc^	50.97 ± 3.21 ^b^
T2	13.18 ± 2.29 ^bc^	25.84 ± 1.34 ^b^	32.33 ± 5.63 ^bc^	28.60 ± 2.43 ^cd^
T3	10.82 ± 1.47 ^c^	17.42 ± 1.08 ^e^	20.02 ± 2.06 ^d^	19.50 ± 0.75 ^d^
T4	14.21 ± 5.78 ^bc^	19.09 ± 1.76 ^d^	27.57 ± 1.97 ^c^	30.80 ± 5.48 ^c^
T5	15.18 ± 1.01 ^b^	25.08 ± 1.34 ^b^	33.62 ± 1.54 ^b^	34.17 ± 3.16 ^c^
T6	20.61 ± 1.54 ^a^	38.31 ± 1.10 ^a^	46.96 ± 2.12 ^a^	92.90 ± 10.86 ^a^
CD	4.24	1.22	5.75	10.53
MSE	5.43	0.45	9.98	33.52
SE(m)	1.35	0.39	1.82	3.34
SE(d)	1.9	0.55	2.58	4.73
CV (%)	15.92	2.70	9.87	13.52

## Data Availability

The original contributions presented in this study are included in the article. Further inquiries can be directed to the corresponding authors.
